# Age-Dependent Variation in Hormonal Concentration and Biochemical Constituents in Blood Plasma of Indian Native Fowl

**DOI:** 10.4061/2010/737292

**Published:** 2010-12-29

**Authors:** Avishek Biswas, Jag Mohan, Kochigant Venkata Hanumant Sastry

**Affiliations:** ^1^Division of Physiology and Reproduction, Central Avian Research Institute, Izatnagar, Bareilly 243122, Uttar Pradesh, India; ^2^Defence Institute of High Altitude Research (DIHAR), DRDO, C/O: 56 APO, Leh 194101, Jammu and Kashmir, India

## Abstract

This experiment was to investigate the age-related changes in hormonal concentration and biochemical constituents of blood plasma in Indian native desi fowl. One hundred and sixty two (54 from each breed, i.e., Kadaknath (KN), Aseel peela (AP), and White leghorn (WLH)) day-old female chicks were randomly divided into nine groups each of 18 chicks (3 groups × 3 replicates). WLH was taken in this study to compare the characteristics of Indian native desi fowl. The highest level of estrogen hormone in WLH and desi fowl in blood plasma was occurred at 18 and 24 wks of age, respectively. Whereas, the peak of progesterone hormone in WLH hens noticed around 24 wks, in case of desi fowls, it was at 30 wks of age. Irrespective of the breed, the hormonal profile of Triiodothyronine (T3) and Thyroxine (T4) in blood plasma was found highest around 6 to 12 wks of age. Activities of acid phosphatase (ACP) increased with the reduction of alkaline phosphate (ALP) activities at different time intervals. Irrespective of the breed, transaminases (glutamic oxaloacetic transaminase (GOT) and glutamic pyruvic transaminase (GPT)) activities of blood plasma increased linearly with the advancement of the age. From this study, it may be concluded that sexual maturity of the Indian native desi fowl occurred nearly 6 wk later (24 wk) than WLH.

## 1. Introduction

According to FAO [1], population of India is expected to increase from 1.03 billion in 2003 to 1.35 billion in 2025. To feed rapid growing of human population and to fight against malnutrition, there is need to produce sufficient amount of meat and eggs. National Advisory Committee has recommended annual consumption of 180 eggs, at the rate of half egg per day per person and 9 kg of poultry meat per year at the rate of approximately 25 gm meat every day. However, at present its availability is only 45 eggs and 1.60 kg of poultry meat, respectively. The wide gap, therefore, exists between demand and supply of eggs and meat for our nutritionally starved masses need to be bridged through poultry in which desi fowl can play a very important role because that is the back bone of our rural poultry production and constituting nearly 70% (Kadaknath, Aseel, etc.). 

Kadaknath (KN) and Aseel Pela (AP) are the Indian native fowl which are well known for poor egg production (120–140 eggs/laying cycle), slow growth, frequent broodiness, smaller body size (1.2–1.5 kg in adult), small egg size (40–42 g), and late sexual maturity. Eggs and meat production in birds is regulated by several complex interactions in which avian reproduction played an important role. This area of science is totally neglected and uninvestigated in desi fowl. The hormonal profile and reproductive development of desi fowl from postnatal to adult stage (age-dependent development) is not known. Knowledge gained from this could be used for neuroendrocrine manipulation, which is a popular tool for improving egg production through hormone administration. It is hypothesized that slow maturation of ovary and oviduct in desi fowl (Kadaknath and Aseel peela) occurs up to 18 to 19 weeks of age.

Numbers of studies have been done to understand the relationship in exotic breed [2–4], but studies on Indian native fowl (KN and AP) are lacking. So, this study described here attempted to know the age-related changes in hormonal concentration and biochemical composition in blood plasma of Indian native fowl.

## 2. Materials and Methods

### 2.1. Experimental Stock

More than one hundred fertile eggs from each breed, that is, Kadaknath (KN), Aseel peela (AP), and White Leghorn (WLH) were procured from the experimental layer farm, and the eggs were hatched at Institute's hatchery. WLH was taken in this study to compare the characteristics of Indian native desi fowl. One hundred and sixty two (54 from each breed) day-old female chicks were randomly divided into nine groups each of 18 chicks (3 groups × 3 replicates). The day-old chicks were housed on floor, covered with litter, and maintained under uniform husbandry conditions. They were given normal rations (21% crude protein, 2,900 Kcal/kg ME, 3% total calcium and 0.5% total phosphorus) as per the requirement of particular age group and water *ad libitum* with a constant light 14 hrs/day throughout the experiment. Mortality as and when occurred is recorded. All the experiments were conducted strictly in accordance with the guideline of  Institutional Animal Ethics Committee (IAEC).

### 2.2. Blood Collection

Blood samples were collected (2 mL) from all the birds through wing vein from 6th week to 30th week. Heparin at the rate of 5 IU/mL was used as anticoagulant. The blood was centrifuged at 2500 rpm for 10 minutes to separate the plasma for estimation of hormones (estrogen, progesterone, Triiodothyronine (T3), and Thyroxine (T4)) and biochemical constituents. Plasma samples were stored at −40°C till use.

### 2.3. Estimation of Hormones

Plasma estrogen, progesterone, T3, and T4 concentration were determined using ELISA kits (Dia Metra, USA). The absorbance was recorded by using *Spectramax*. The hormones concentration was determined by plotting OD of samples against the standard graph. Performed the hormone examination in all the three breeds, that is, KN, AP, and WLH in every six wks interval from 6th to 30th wks.

### 2.4. Estimation of Phosphatases and Transaminases

Acid phosphatase (EC 3.1.3.2, ACP) was analysed by using the method as described by Andersch and Szcypinski [5] and Alkaline phosphatase (EC 3.1.3.1, ALP) was examined by method of Bessay and Lowry [6]. Glutamic oxaloacetic transaminase (EC 2.6.1.1 GOT) and Glutamic pyruvate transaminase (EC 2.6.1.2, GPT) were estimated by Reitman and Frankel [7] method by using the kits supplied by Qualigens Diagnostics.

### 2.5. Statistical Analysis

Data were analyzed using statistical software package developed at the Computer Centre of Central Avian Research Institute and followed standard procedures for ANOVA [8] and Duncan's multiple range tests [9] by comparing means for significant differences.

## 3. Results

### 3.1. Hormone Concentration

The age-dependent variation in the level of estrogen, progesterone, T_3_ and T4 concentration in blood plasma of different breeds of fowl is presented in Tables 1, 2, 3, 4, and 5. The estrogen profile of blood plasma in WLH hen increased significantly (*P* < .05) and rapidly with age until 18 wks and decreased subsequently. In contrast, the level of progesterone was increased slowly by the end of 18 wks and significantly (*P* < .05) increased rapidly in the following intervals then remain constant. In Indian native fowl, the peak of estrogen hormone exhibited nearly 6 wk later (24 wks) than WLH. After 24 wks the reduction of estrogen level associated with increase in the level of progesterone. Within the desi fowl at all intervals the hormonal profile of estrogen and progesterone was found higher in KN than AP. Both the hormones T3 and T4 have been examined in present study in relation to age advancement associated with growth of chicken. Irrespective of breeds, the concentration of  T4 was found highest around the age of 6 wks but declined significantly (*P* < .05) until the age of 12 wks. Subsequently, T4 concentration remained relatively stable during the following week's intervals. Within breeds, maximum profile of T4 was examined in AP followed by KN and WLH at various intervals. However, in case of T3, within the breed, no significant difference was found throughout the study at specific interval.

### 3.2. Phosphatases and Transaminases Activities

The phosphatases (ACP and ALP) and transaminases (GOT and GPT) activities in the blood plasma of different breeds are presented in Tables 1 to 5. With the advancement of age, a reverse trend was found in both the phosphomonoesterase (ACP and ALP) activities. ACP activity increases significantly (*P* < .05) with age whereas a reduction (*P* < .05) in the activity of ALP was accounted. Highest ACP activity was revealed by KN followed by WLH and AP. Regarding the ALP, the highest specific activity was expressed by the AP rather than KN and WLH. Activity of both the transaminases increased linearly throughout the study period, irrespective of the breed. Mean values of GOT of WLH at the age of 6 and 12 wk was significantly (*P* < .05) higher than two desi breeds (i.e., KN and AP) whereas the mean values of GPT of WLH remained significantly (*P* < .05) higher at all the intervals of the study period that is, 6, 12, 18, 24, and 30 wks when compared with other breeds.

## 4. Discussion

### 4.1. Hormone Concentration

The data reveals that the age around 18 wks is the transition phases, which correlates the age of initiation of sexual maturity in WLH. The similar pattern of progesterone profile was recorded by Williams and Sharp [10] in WLH hens. Below this or around this age the ovary is expected to have the large number of follicles below 10 mm in size, which secretes the large quantity of estrogen from theca layer. After 18th wks of age, follicles mature continuously and they grow from less than 1 mm to approximately 35–40 mm in diameter. They are recruited in to the yolk-filled hierarchy when they have reached approximately 10 mm or above in size. Before recruitment of follicles in the yolk filled hierarchy (below 10 mm size of follicles or around 18 wks of age), they produce large quantities of various hormones such as dehydro epiandrosterone, androstenedione, and estrogen. After recruitment of follicles in the yolk-filled hierarchy, they produce decreasing quantities of estrogens and gain the capacity to produce progesterone. After progressing through the yolk-filled hierarchy with advancement of age, the largest ovarian follicular loses the ability to produce androgens and estrogens and secrets the large quantities of progesterone in the systemic circulation [11]. This might be the possible reason in our study, which causes the drastic reduction in estrogen level after 18 wks in WLH hens and simultaneously significant (*P* < .05) increase in the level of progesterone in blood circulation (Tables 1–5). 

The sexual maturity in desi fowl occurred late as evidenced by peak of estrogen level exhibited nearly 6 wk later (24 wk) than WLH. This may be due to breed differences, which vary from breed to breed. After 24 wks, the reduction of estrogen concentration is associated with increase in the level of progesterone (Tables 4 and 5). This preovulatory surge of progesterone stimulates the release of LH, which in turn causes ovulation of the largest follicle. Besides ovulation, LH stimulates steroidogenesis in ovary and causes the production of androgens and estrogens that stimulate reddening of comb, deposition of medullary bone, formation of oviduct, and production of the yolk precursors by the liver [11–13]. Within the desi fowl, at all intervals, the hormonal profile of both the hormones was found higher in KN than AP. This may be related with early sexual maturity (2-3 wk) in KN.

The concentration of T3 hormone was also noticed maximum in early ages (6–12 wks). Thereafter, a linear and significant (*P* < .05) decrease was recorded till the end of experiment (30 wks) in all the breeds. These results indicated that T3 hormones expressed prominent role in growth and development of birds as evidenced by significant reduction of its concentration with age as compared to T4 hormone. It is well known that T3 plays a far greater role in bio-oxidation process in cells than does T4 [14]. Almost identical effects of T3 and T4 in birds were observed by Shellbarger [15], which is not the case in mammals. Bobek et al. [14] later investigated that T3 is the main thyroid hormones regulating oxygen consumption particularly in young chickens. Klandorf et al. [16], finally confirmed that T3 in chickens is a metabolically more active substance than T4. Thyroxine constitutes approximately 60% and T3 approximately 40% of the circulating thyroid hormone in the domestic birds [17]. Nevertheless, the higher profile of both hormones during early 6 wks of age may be reflected to an increased metabolic rate, especially to energy production as well as to their involvement in the growth and development of the chickens at early age of life. 

It is well documented that thyroid hormone T3 and T4 are involved in wide range of metabolic activities influencing the growth and development of birds. The thyroid hormones are primarily involved in energy production by increasing the metabolic rate in turn heat production. The importance of these T3 and T4 hormones to the growth and development of organism is most visible in deficient animals that exhibit stunted growth and lower productivity. Since the production of broilers in poultry industry lasts only 6 wks, now one would expect that how important are the T3 and T4 in the growth and development of chicken [18]. 

To compare the age-dependent variation in the mean values of T3 and T4 in different breeds of chicken, we do not have data in the literature to compare with others. However, Stojević et al. [18], reported the age-dependent (3 to 6 wk) increase in the concentration of T3 and T4 hormone. As available literature is more scarce, therefore, this study suggested conducting more planned investigations on T3 and T4 hormone in view of their importance in poultry production.

### 4.2. Phosphatases and Transaminases Activities

The ALP activity in blood commonly used as an aid to the diagnosis of bone diseases and obstructive jaundice. In poultry, the main objective for the study of this enzyme was to find out the possible relationship between enzyme activity and various economic traits [19–22]. In our study, the activity of ALP decreased significantly (*P* < .05) with the increase of age from 6 to 24 wks. The increase of age is associated with economic traits (egg production), where the activity of ALP decreased as evidenced by the results presented in Tables 1–5. This variation in enzyme activities may be due to breed differences, which reflect variation in the overall metabolic rate in different breeds. This indicated that ALP activity is negatively correlated with economic traits. These results find the support from the work carried out by A. Mazumder and N. Mazumder [23] in WLH breed in which the activity of ALP decreased from 62.85 K.A. units (12 wks) to 25.80 K.A. units (24 wks) with the advancement of the age. The present finding is also in agreement with other workers [20, 24]. In contrast, several workers reported that higher ALP activity is associated with higher productivity [25]. Foregoing discussion on the possible association between ALP activity and various economic traits in poultry revealed that the findings are contradictory indicating more investigations are needed. These results suggested that the activity of GOT and GPT is positively correlated with age. This correlation of these enzyme systems with age in the body might be as per the requirement of development of various biological systems in the body including reproductive system. On both the GOT and GPT enzymes in fowl, we do not have data for comparison with others.

## 5. Conclusion

From the ongoing discussion, it may be concluded that sexual maturity of the Indian native desi fowl occurred nearly 6 wk later (24 wk) than WLH (18 wk).

## Figures and Tables

**Table 1:** Age-dependent variation in hormonal concentration and biochemical constituents of blood plasma (6th wk) in different breeds of Indian native hen (Mean ± S.E.M, *n* = 6).

Group	Estrogen (pg/mL)	Progesterone (ng/mL)	T3 (ng/mL)	T4 (ng/mL)	ACP (*μ*mole/min./mg protein)	ALP (*μ*mole/min./mg protein)	GOT (*n* mole/min./mg protein)	GPT (*n* mole/min./mg protein)
WLH	10.28 ± 0.26 NS	0.65^b^ ± 0.03	4.83 ± 0.16 NS	16.25^a^ ± 1.92	0.65^a^ ± 0.16	40.49^a^ ± 4.20	30.38^b^ ± 2.09	2.27^b^ ± 0.65
KN	9.84 ± 0.43 NS	0.46^a^ ± 0.06	4.91 ± 0.40 NS	17.73^ab^ ± 1.12	1.78^b^ ± 0.40	52.50^b^ ± 3.69	23.29^a^ ± 1.14	1.26^a^ ± 0.45
AP	9.28 ± 0.24 NS	0.42^a^ ± 0.09	4.92 ± 0.34 NS	18.80^b^ ± 1.76	0.67 ± 0.11	64.60^c^ ± 3.08	23.17^a^ ± 2.11	1.11^a^ ± 0.23

Mean values bearing the same superscripts in a column do not differ significantly (*P* < .05).

NS: nonsignificant.

**Table 2:** Age-dependent variation in hormonal concentration and biochemical constituents of blood plasma (12th wk) in different breeds of Indian native hen (Mean ± S.E.M, *n* = 6).

Group	Estrogen (pg/mL)	Progesterone (ng/mL)	T3 (ng/mL)	T4 (ng/mL)	ACP (*μ*mole/min./mg protein)	ALP (*μ*mole/min./mg protein)	GOT (*n* mole/min./mg protein)	GPT (*n* mole/min./mg protein)
WLH	45.34^b^ ± 3.31	0.70^b^ ± 0.04	4.72 ± 0.15 NS	13.15^ab^ ± 1.84	1.25^b^ ± 0.19	40.31^a^ ± 4.19	35.69^b^ ± 1.06	3.79^b^ ± 0.35
KN	34.32^a^ ± 4.28	0.69^b^ ± 0.04	4.82 ± 0.40 NS	14.31^b^ ± 1.28	2.09^c^ ± 0.25	52.10^b^ ± 2.14	27.80^a^ ± 2.17	1.73^a^ ± 0.28
AP	32.77^a^ ± 1.16	0.55^a^ ± 0.05	4.91 ± 0.33 NS	12.49^a^ ± 1.29	0.80^a^ ± 0.12	65.22^c^ ± 2.70	27.01^a^ ± 2.34	1.63^a^ ± 0.12

Mean values bearing the same superscripts in a column do not differ significantly (*P* < .05).

NS: nonsignificant.

**Table 3:** Age-dependent variation in hormonal concentration and biochemical constituents of blood plasma (18th wk) in different breeds of Indian native hen (mean ± S.E.M, *n* = 6).

Group	Estrogen (pg/mL)	Progesteron (ng/mL)	T3 (ng/mL)	T4 (ng/mL)	ACP (*μ*mole/min./mg protein)	ALP (*μ*mole/min./mg protein)	GOT (*n* mole/min./mg protein)	GPT (*n* mole/min./mg protein)
WLH	133.27^c^ ± 2.29	1.33 ± 0.27 NS	2.69 ± 0.59 NS	11.97^a^ ± 0.73	1.58^ab^ ± 0.26	40.69^a^ ± 4.09	37.79 ± 1.49 NS	5.59^c^ ± 0.46
KN	68.97^b^ ± 2.80	1.23 ± 0.22 NS	2.85 ± 0.36 NS	13.51^ab^ ± 0.84	3.48^b^ ± 0.19	43.48^a^ ± 3.82	35.42 ± 1.16 NS	3.64^b^ ± 0.26
AP	3.65^a^ ± 1.88	1.10 ± 0.48 NS	3.14 ± 0.36 NS	14.90^b^ ± 1.20	1.22^a^ ± 0.16	54.92^b^ ± 4.25	31.62 ± 2.08 NS	1.35^a^ ± 0.83

Mean values bearing the same superscripts in a column do not differ significantly (*P* < .05).

NS: nonsignificant.

**Table 4:** Age-dependent variation in hormonal concentration and biochemical constituents of blood plasma (24th wk) in different breeds of Indian native hen (mean ± S.E.M, *n* = 6).

Group	Estrogen (pg/ml)	Progesterone (ng/mL)	T3 (ng/mL)	T4 (ng/mL)	ACP (*μ*mole/min./mg protein)	ALP (*μ*mole/min./mg protein)	GOT (*n* mole/min./mg protein)	GPT (*n* mole/min./mg protein)
WLH	114.03^a^ ± 2.34	3.75^ab^ ± 0.37	1.23 ± 0.20 NS	11.46^a^ ± 1.08	1.93^a^ ± 0.32	22.46^a^ ± 2.38	48.08 ± 2.17 NS	16.96^b^ ± 1.20
KN	134.28^c^ ± 2.87	3.92^b^ ± 0.56	1.48 ± 0.36 NS	12.35^a^ ± 0.90	3.79^b^ ± 0.22	30.82^b^ ± 1.64	46.70 ± 1.75 NS	9.87^a^ ± 0.96
AP	126.07^b^ ± 2.60	2.90^a^ ± 0.71	1.52 ± 0.50 NS	14.23^b^ ± 1.34	1.57^a^ ± 0.26	36.21^c^ ± 2.78	44.00 ± 1.18 NS	7.50^a^ ± 0.53

Mean values bearing the same superscripts in a column do not differ significantly (*P* < .05).

NS: nonsignificant.

**Table 5:** Age-dependent variation in hormonal concentration and biochemical constituents of blood plasma (30th wk) in different breeds of Indian native hen (mean ± S.E.M, *n* = 6).

Group	Estrogen (pg/mL)	Progesterone (ng/mL)	T3 (ng/mL)	T4 (ng/mL)	ACP (*μ*mole/min./mg protein)	ALP (*μ*mole/min./mg protein)	GOT (*n* mole/min./mg protein)	GPT (*n* mole/min./mg protein)
WLH	79.44^a^ ± 9.81	3.95^a^ ± 0.19	0.86 ± 0.10 NS	13.79 ± 1.21 NS	3.31^b^ ± 0.22	20.76^a^ ± 5.13	52.15 ± 1.49 NS	19.66^c^ ± 1.65
KN	107.70^c^ ± 3.90	4.50^b^ ± 0.51	0.98 ± 0.08 NS	14.04 ± 0.91 NS	3.97^b^ ± 0.16	28.11^ab^ ± 2.40	51.82 ± 1.05 NS	14.06^b^ ± 0.95
AP	96.56^b^ ± 4.94	3.64^b^ ± 0.32	0.99 ± 0.23 NS	14.60 ± 0.81 NS	2.78^a^ ± 0.22	32.10^b^ ± 3.37	50.46 ± 0.75 NS	9.78^a^ ± 1.15

Mean values bearing the same superscripts in a column do not differ significantly (*P* < .05).

NS: nonsignificant.

## References

[1] FAO (2003). *Quarterly Bulletin of Statistics*.

[2] Swain BK, Johri TS, Majumdar S (2000). Effect of supplementation of vitamin E, selenium and their different combinations on the performance and immune response of broilers. *British Poultry Science*.

[3] Surai PF (2002). Selenium in poultry nutrition 1. Antioxidant properties, deficiency and toxicity. *World’s Poultry Science Journal*.

[4] Lin YF, Chang SJ, Yang JR, Lee YP, Hsu AL (2005). Effects of supplemental vitamin E during the mature period on the reproduction performance of Taiwan Native Chicken cockerels. *British Poultry Science*.

[5] Andersch MA, Szcypinski AJ (1947). Use of P-nitrophenyl phosphate as substrate in determination of serum acid phosphatase. *American Journal of Clinical Pathology*.

[6] Bessay OA, Lowry OH (1946). A method for rapid determination of alkaline phosphatase with 5 cubic millimeters of serum. *Journal of Biological Chemistry*.

[7] Reitman S, Frankel S (1957). A calorimetric method for determination of glutamic oxaloacetic and glutamic pyruvic transaminase activities. *Journal of Dairy Science*.

[8] Snedecor GW, Cochran WG (1994). *Statistical Methods*.

[9] Duncan B (1955). Multiple range and multiple F tests. *Biometrics*.

[10] Williams JB, Sharp PJ (1977). Comparison of plasma progesterone and luteinizing hormone in growing hens from eight weeks of age to sexual maturity. *Journal of Endocrinology*.

[11] Etches RJ (1990). The ovulatory cycle of the hen. *CRC Critical Review of Poultry Biology*.

[12] Sturkie PD (1965). *Avian Physiology*.

[13] Hafez ESE (1995). *Reproduction in Farm Animals*.

[14] Bobek S, Jastrzebski M, Pietras M (1977). Age related changes in oxygen consumption and plasma thyroid hormone concentration in the young chicken. *General and Comparative Endocrinology*.

[15] Shellbarger CJ (1955). A comparison of triiodothyronine and thyroxin in chick goiter prevention test. *Poultry Science*.

[16] Klandorf H, Sharp PJ, Newcomer WS (1981). The influence of feeding patterns on daily variations in the concentrations of plasma thyroid hormones in the hen. *IRCS Medical Science*.

[17] Wentworth BC, Mellen WJ (1961). Circulating thyroid hormones in domestic birds. *Poultry Science*.

[18] Stojević Z, Milinković-Tur S, Ćurčija K (2000). Changes in thyroid hormones concentrations in chicken blood plasma during fattening. *Veterinarski Arhiv*.

[19] Jain AK, Joshi SC, Rawat JS, Pandey MD (1976). Serum alkaline phosphatase isozymes and their relationship with certain economic traits in poultry. *Indian Journal of Animal Science*.

[20] Kumar A, Rawat JS (1975). Effect of age, sex and reproduction on serum enzymes and electrolyte levels in White Leghorn birds. *Indian Journal of Animal Science*.

[21] Chowdhury RP (1972). Polymorphism and its application in poultry breed. *Indian Poultry Gazette*.

[22] Smith JL, Goodman BL (1970). Heritability and correlation estimates for serum alkaline phosphatase and growth in the chick. *Poultry science*.

[23] Mazumder A, Mazumder NK (1982). Effect of storage conditions, age, sex, strain and breed on the plasma ALP activity in poultry. *Indian Journal of Animal Science*.

[24] Tamaki Y, Tanabe Y (1970). Genetic control of multiple molecular forms of the alkaline phosphatase in chicken plasma. *Poultry science*.

[25] Wilcox FH (1966). A recessively inherited electrophoretic variant of alkaline phosphatase in chicken serum. *Genetics*.

